# Preoperative Chemoradiotherapy for Locally Advanced Rectal Cancer: A Retrospective Analysis

**DOI:** 10.7759/cureus.81427

**Published:** 2025-03-29

**Authors:** Jacob F Wahba, Gregory Knight, Mohamed Husein, Bogdan Paun, Darin Gopaul

**Affiliations:** 1 Faculty of Health Sciences, Wilfrid Laurier University, Waterloo, CAN; 2 Department of Oncology, Grand River Regional Cancer Centre, Grand River Hospital, Kitchener, CAN; 3 Department of Surgery, Grand River Hospital, Kitchener, CAN; 4 Department of Radiation Oncology, Grand River Regional Cancer Centre, Grand River Hospital, Kitchener, CAN

**Keywords:** chemoradiotherapy (chemo-rt), colorectal cancer, distant metastases, locally advanced rectal cancer, neoadjuvant therapy, oncological outcomes, pathologic complete response, rectal cancer surgery, survival analysis

## Abstract

Purpose

To evaluate the outcomes of patients with locally advanced rectal cancer (LARC) treated with preoperative chemoradiotherapy (CRT) at a community cancer center.

Methods

A retrospective chart review was conducted for patients with biopsy-proven rectal adenocarcinoma treated with CRT between January 2017 and June 2020. Patients were excluded if there was metastatic disease (stage IV) at presentation, if curative resection was not planned, or if they received additional preoperative chemotherapy. Preoperative radiotherapy was typically 50.4 Gy in 28 fractions with concurrent capecitabine chemotherapy, followed by surgery six to eight weeks later. Postoperative adjuvant FOLFOX chemotherapy was typically recommended in suitable patients. Outcomes measured included surgical margin status, pathological complete response (pCR), local recurrence rate, distant metastases, cancer-specific survival, and overall survival.

Results

A total of 120 patients underwent preoperative CRT during this period. Seven patients did not undergo subsequent surgical resection. The pCR rate was 14%, and R0 resection (negative margins) was achieved in 93% of cases. The cumulative incidence of local recurrence was 6%, and distant metastases developed in 23% of patients. The most common metastatic sites were the liver and lungs. With a median follow-up of 28 months, Kaplan-Meier analyses demonstrated a 78% cancer-specific survival (CSS) and 75% overall survival (OS).

Conclusion

Preoperative CRT resulted in a 14% pCR rate, which was associated with high R0 (93%) and low local recurrence rates (6%). Distant metastatic recurrence rate remains a concern (23%).

## Introduction

Rectal cancer is the third most common cancer worldwide. Despite advancements in screening, prevention, and treatment, rectal cancer continues to be a leading cause of cancer-related morbidity and mortality [1]. The German CAO/ARO/AIO-94 study conducted a randomized trial comparing preoperative chemoradiotherapy (CRT) and postoperative CRT. This study demonstrated preoperative CRT significantly improved local control (7.1% vs 10.1%, respectively) with lower treatment-related toxicity. No improvement in overall survival (OS) was demonstrated [2]. The goal of this retrospective study is to determine the outcome of rectal cancer patients in a more contemporary cohort treated with preoperative CRT at a community cancer center.

## Materials and methods

A retrospective chart review was conducted for patients with biopsy-proven rectal adenocarcinoma treated with conventionally fractionated preoperative CRT at the Grand River Regional Cancer Centre (GRRCC) from January 2017 to June 2020. The study period of January 2017 to June 2020 was chosen due to a policy shift in mid-2020, due to the COVID-19 pandemic, which resulted in a large decrease in the use of preoperative chemoradiotherapy and an increase in the adoption of the Rectal cancer And Preoperative Induction therapy followed by Dedicated Operation (RAPIDO) approach. This period acts as a baseline for comparison before the shift in treatment approach. Patients were excluded if there was metastatic disease (stage IV) at presentation, if curative resection was not planned or if they received additional preoperative chemotherapy. A query of the radiotherapy database identified 145 patients that were treated with preoperative CRT from January 2017 to June 2020. A total of 25 patients were excluded from the outcome analysis (11 with stage IV cancer, four with recurrent cancers, and two treated with additional preoperative chemotherapy). Eight additional patients were excluded because they underwent surgery at another hospital, and no surgical or pathological information was available.

All patients were evaluated endoscopically, as well as with CT of the chest, abdomen and pelvis (CT CAP), and MRI of the pelvis (unless contraindicated). TNM (Tumor (T), Nodes (N), and Metastasis (M)) staging was performed according to the American Joint Committee on Cancer (AJCC 8th edition, 2017) [3]. Pelvic radiotherapy consisted of 50.4 Gy in 28 fractions typically with a field size reduction off the small bowel was at 45 Gy and using a three-field 3D conformal technique. Capecitabine chemotherapy (825 mg/m^2^) was administered orally twice daily on radiotherapy days. Curative intent surgery was planned six to eight weeks after treatment completion. Post operatively, adjuvant FOLFOX chemotherapy was typically recommended for suitable patients. Our follow up protocol follows the Cancer Care Ontario (CCO) guidelines. Following the completion of adjuvant chemotherapy, patients undergo a medical history, physical exam, and carcinoembryonic antigen (CEA) every six months for five years, and a CT CAP annually for three years. Colonoscopy is performed one year after surgery and then every five years if findings are normal.

Patient follow-up information was gathered from the hospital electronic medical record. Outcome was evaluated by surgical margin status, type of surgery, pathologic complete response (pCR), local recurrence rate (LRR), distant metastases (DM), cancer specific survival (CSS), and overall survival (OS). Local recurrence was defined as any pelvic recurrence, detected by imaging and/or biopsy confirmation. DM was detected by cross-sectional imaging and confirmed by biopsy when feasible. CSS was measured from the start of CRT until death from rectal cancer or the date of last follow-up. OS was measured from the start of CRT until death from any cause or the date of last follow-up. Cumulative incidence was used to estimate LRR and DM, and the Kaplan-Meier method was used to assess CSS and OS. This study was approved by our institutional research ethics board.

## Results

Patient and tumor characteristics

One hundred and twenty patients were planned for preoperative CRT. The average age was 61 years (range: 28-85 years). Patient and tumor characteristics are summarized in Table 1. Most patients had cT3/T4 or node-positive disease. Additionally, 27% of the patients were diagnosed with cT4 tumors, and 73% had nodal involvement. Fourteen patients (12%) were cT2/earlyT3 N0, and one patient had a cT2N0 tumor, which was 3.5 cm from the anal verge and 2 mm from the mesorectal fascia. Moreover, 23% of tumors were situated within 5 cm from the anal verge.

**Table 1:** Baseline characteristics and clinical staging of the 120 patients eligible for preoperative chemoradiotherapy

Eligible patients	Patients
Age–Years (range)	61 (range 28-85)
Clinical Tumor Stage – no. (%)	
T2	8 (6.7)
T2/Early T3	30 (25.0)
T3	41 (34.2)
T3/Possible T4	8 (6.7)
T4	32 (26.7)
Unknown	1 (0.8)
Clinical Node Stage – no. (%)	
N0	33 (27.5)
N1	79 (65.8)
N2	8 (6.7)
Distance from Anal Verge – no. (%)	
<5 cm	28 (23.3)
5-10 cm	45 (37.5)
>10 cm	29 (24.2)
Unknown	18 (15.0)

Three patients were not able to complete CRT due to treatment-related toxicity, including one treatment-related mortality (febrile neutropenia and sepsis). The average follow-up at the cancer centre was 28 months (range: 3-88 months). Some patients were discharged for follow-up at one of our satellite oncology clinics and referred to the GRRCC only if their disease recurred.

Seven patients did not proceed with curative intent surgery. One patient exhibited a complete clinical response and was managed with a watch-and-wait strategy, remaining cancer-free after 44 months. Two patients developed unresectable metastatic disease, and surgery was aborted. One patient was deemed unresectable due to a tumor fixed to the pelvic sidewall. One patient died prior to surgery due to treatment-related toxicity. One patient with Crohn’s disease had CRT discontinued after 13 fractions and was deemed too frail for surgery. One patient was hospitalized after 26 fractions due to severe colitis symptoms and a pulmonary embolism and also did not proceed to surgery.

Pathological findings and outcomes

Among the 120 patients, 113 (94%) underwent curative-intent surgery with a mean of seven weeks (4-12 weeks) after preoperative CRT (Table 2). Seventy-four patients (65%) had a low anterior resection (LAR), 37 patients (33%) an abdominoperineal resection (APR), and two patients (2%) had a complete proctocolectomy. Four of the 18% of ypT0 patients had residual nodal involvement, reducing the pathological complete response (pCR) rate to 14% (Table 2). Meanwhile, 93% (105 patients) had negative margins, defined as the tumor more than 1 mm from the surgical margin. Two patients (2%) had a tumor at the surgical margin (“ink on tumor”). Both later developed local recurrence and subsequently died of cancer. In the six remaining patients, the margins are reported as positive (less than 1 mm), but there was no tumor noted at the margin. One of these six patients developed a local recurrence, and three have died (two from metastatic rectal cancer and one from esophagus cancer).

**Table 2:** Surgical and pathological outcomes of patients following preoperative chemoradiotherapy(n=113 surgeries) LAR=Low anterior resection; APR=Abdominoperineal resection

Variable	n (%)
Type of Resection (n=113)	
LAR	74 (65.5)
APR	37 (32.7)
Total Proctocolectomy	2 (1.8)
Differentiation Grade: (n=113)	
Well-Differentiated	23 (20.4)
Moderately Differentiated	40 (35.3)
Poorly Differentiated	18 (15.9)
Not Assessed	32 (29.2)
Pathological T Stage (n=113)	
ypT0	20 (17.7)
ypT1	4 (3.5)
ypT2	22 (19.5)
ypT3	49 (43.3)
ypT4	16 (14.2)
Unknown	2 (1.8)
Pathological N Stage (n=113)	
ypN0	81 (71.7)
ypN1	30 (26.5)
ypN2	2 (1.8)

Cumulative incidence of local failure and distant metastases

**Figure 1:**
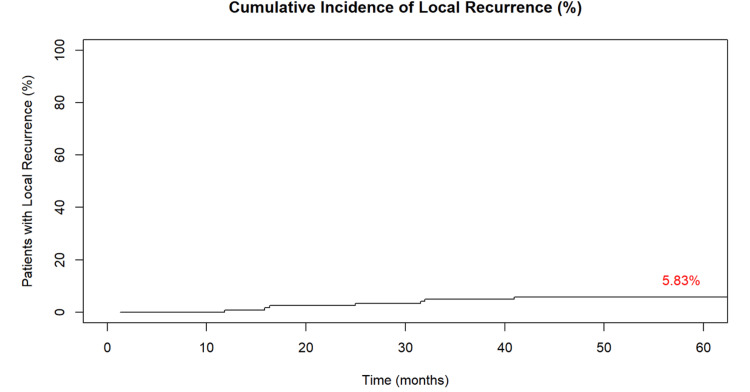
Cumulative incidence of local recurrence in patients over time following preoperative chemoradiotherapy

The cumulative incidence of local recurrence was 6% (seven patients), with a mean time to recurrence of 21 months (range: 9-36 months) (Figure 1). Table 3 shows all patients with local recurrences’ preoperative and postoperative characteristics. Nearly half of the patients (three patients) had positive margins, and four patients had a pathological tumor staging of ypT4. All patients with local failure subsequently died despite salvage efforts.

**Table 3:** Clinical and pathological characteristics of patients with local recurrence LR=Local recurrence; mo=months

Patient No	Distance from Anal Verge (cm)	Clinical Staging	Type of Surgery	Pathological Staging	Surgical Margins (mm)	Time Diagnosed with LF after Surgery	Did Patient also Have Distant Metastases (location)	Salvage Therapy	Prognosis
1	8	T2/3N1	LAR	ypT3N1c	Positive (0)	12 mo	Yes	Chemotherapy	38 mo dead
2	8	T2N1	APR	ypT4bN1b	Positive (0)	9 mo	No	Chemotherapy	48 mo dead
3	8	T2/3N1	LAR	ypT4aN1	Positive (<1)	28 mo	Yes	Pelvis irradiation, chemotherapy, en bloc APR and radical Prostatectomy	48 mo dead
4	4	T3N1	LAR	ypT2N0	Negative (15)	12 mo	Yes	Chemotherapy	44 mo dead
5	8.5	T4N0	LAR	ypT4aN1c	Negative (7)	36 mo	Yes	Chemotherapy	52 mo dead
6	3	T2N0	LAR	ypT1N0	Negative (14)	28 mo	No	None	49 mo dead
7	5.5	T4bN1	APR	ypT4bN0	Negative (3)	19 mo	No	En bloc resection of pelvic tumor with hysterectomy and vaginectomy	41 mo dead

**Figure 2:**
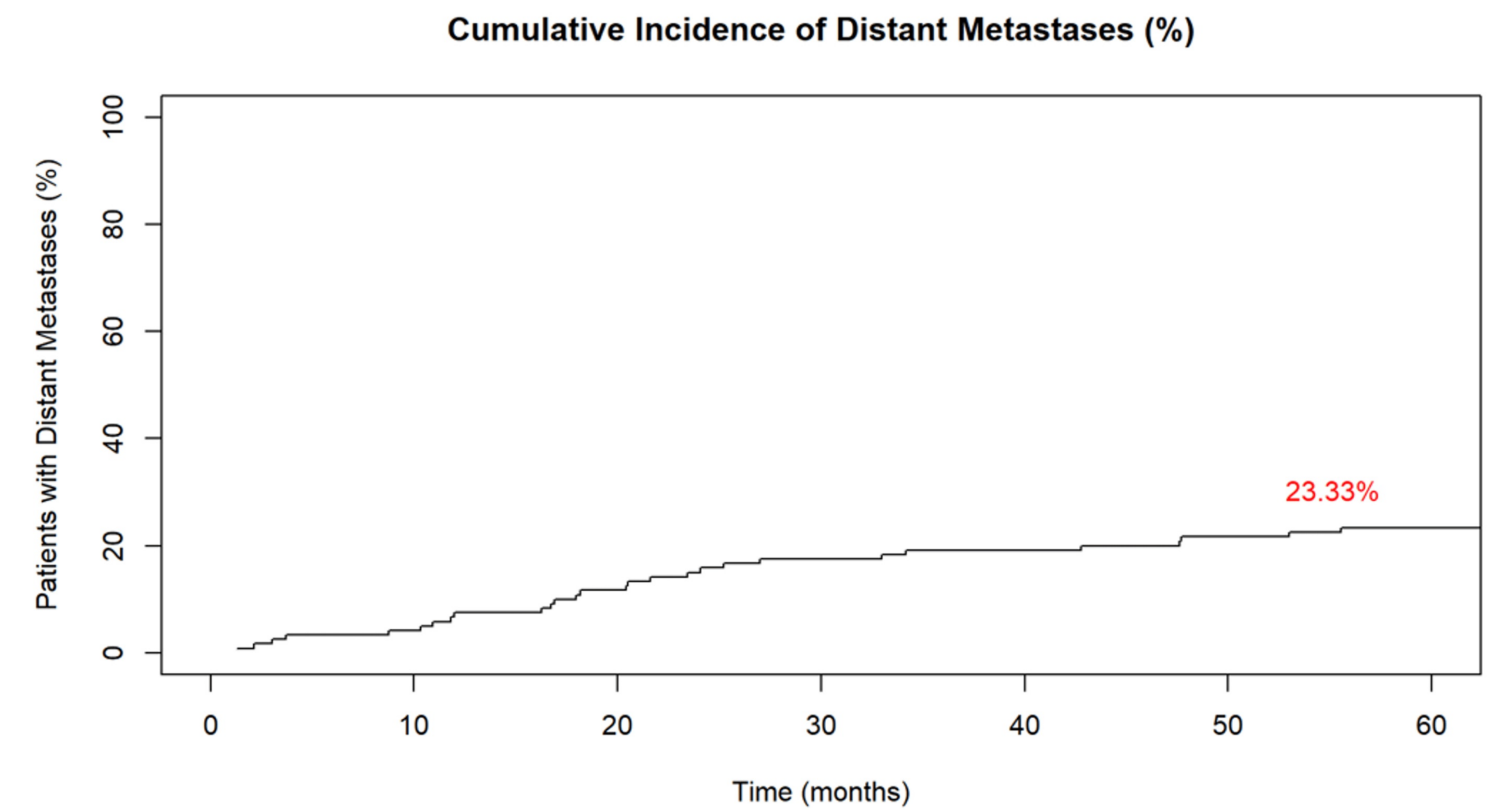
Cumulative incidence of distant metastases among rectal cancer patients after preoperative chemoradiotherapy

The cumulative incidence of distant metastases was 23% (28 patients) (Figure 2). The liver was the most common site of metastasis, followed by the lungs. Additionally, 70% (20 patients) achieved a sustained response with salvage therapy. Salvage therapy typically consisted of chemotherapy and/or surgical resection (e.g., lobectomy, hepatectomy, small bowel resection).

Overall and cancer-specific survival

**Figure 3:**
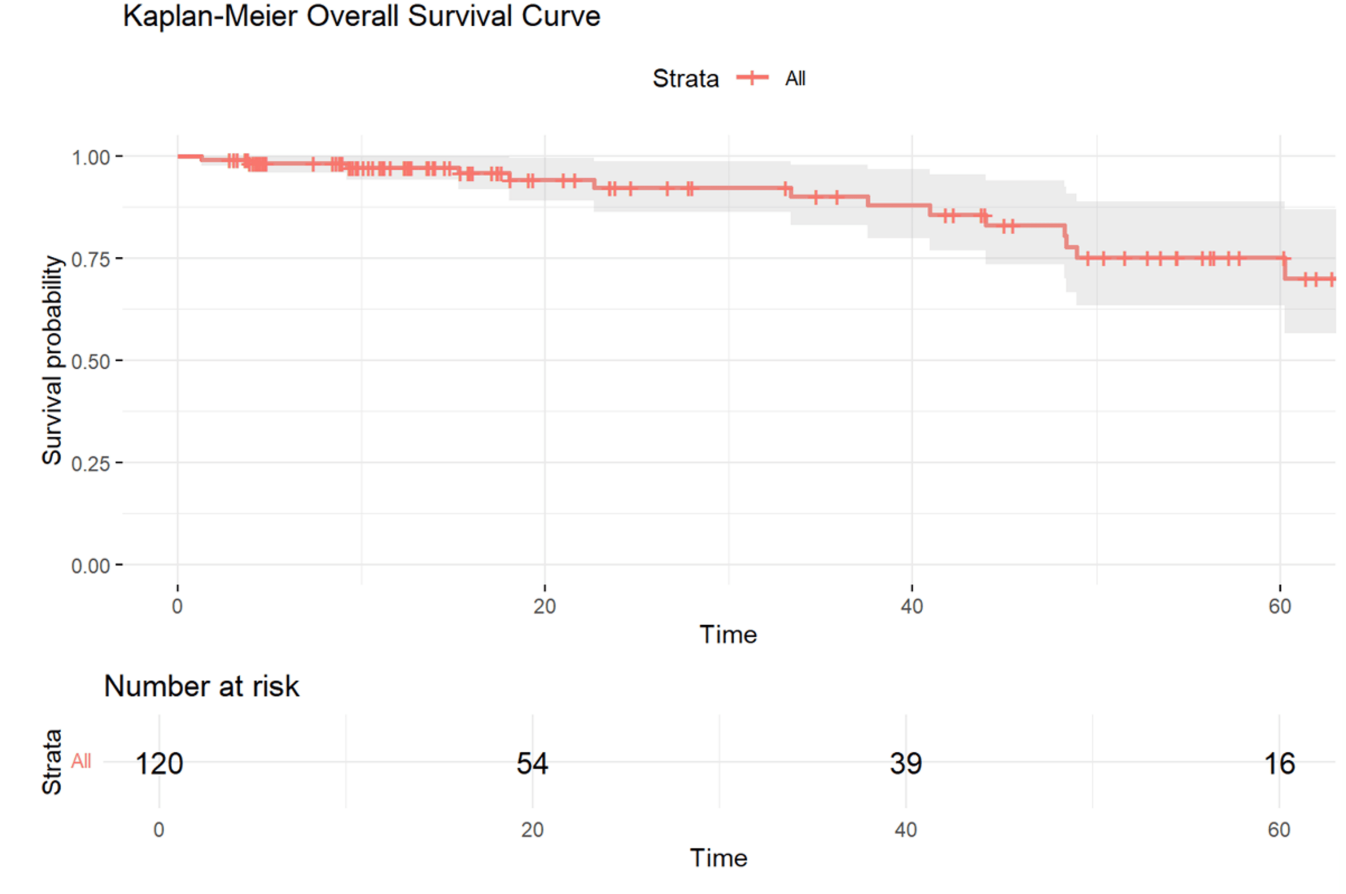
Kaplan-Meier Curve for Overall Survival of Rectal Cancer Patients Following Chemoradiotherapy

**Figure 4:**
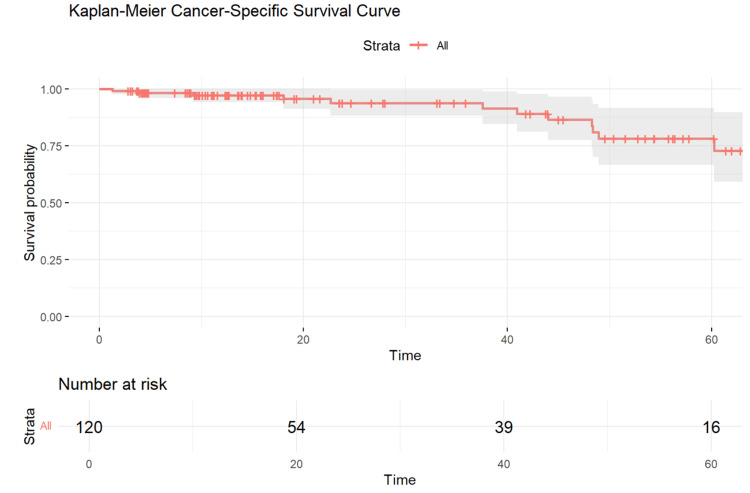
Kaplan-Meier curve for cancer-specific survival of rectal cancer patients following chemoradiotherapy

After a mean follow-up of 28 months (range: 3-83 months), Kaplan-Meier analyses showed an overall survival (OS) of 75% (Figure 3) and a cancer-specific survival (CSS) of 78% (Figure 4).

## Discussion

In our retrospective analysis, preoperative CRT was associated with a low incidence of positive margins (7%) and local failure (6%). This is consistent with previously reported data showing improved local control with preoperative treatment strategies [2,4-6]. The low incidence of local failure is encouraging despite a significant proportion (33%) of patients with clinical T4 or possible T4 disease. The incidence of metastatic recurrence (23%) remains a concern, although relatively lower than other studies [2,4-6,7]. While direct comparisons are difficult, this could be due to shorter follow-up or perhaps improved results with postoperative FOLFOX chemotherapy being offered more routinely during this timeframe compared to single-agent 5-FU in earlier studies. Table 4 summarizes significant findings from clinical trials on preoperative CRT for locally advanced rectal cancer (LARC), including pCR rates, LRR, DM, and OS rates.

**Table 4:** Comparative outcomes from key clinical trials on preoperative chemoradiotherapy in rectal cancer * Refers to the current study

Study (Year)	Number of Patients	Local Recurrence Rate	Overall Survival Rate	Distant Metastasis Rate	pCR Rate
CAO/ARO/AIO-94 (2004) [2]	404	10-year: 7.1%	10-year: 59.6%	29.8%	9%
Bujko et al. (2006) [4]	157	4-year: 14.2%	4-year: 66.2%	34.6%	16.1%
FFCD 9203 (2006) [6]	375	5-year: 8.1%	5-year: 67.4%	28.5%	11.40%
TROG 01.04 (2012) [5]	163	3-year: 4.4%	5-year: 70%	30%	15%
Wahba et al. (2025)*	117	5-year: 5.83%	5-year: 74.61%	23.3%	14.2%

Our study is limited by a short follow-up period for some patients, even though the last patient was treated nearly five years ago. Some patients treated with radiotherapy at our regional cancer center went on to receive surgery and chemotherapy at other hospitals, and we were unable to acquire their records for this retrospective review. This could have resulted in an underestimation of local recurrence due to insufficient follow-up. While most patients with recurrent tumors were likely sent back to our center for palliative radiation, cases addressed completely at other institutions may not have been documented. Furthermore, the absence of access to external databases restricts full follow-up. The use of existing medical records in this retrospective study may have introduced selection bias, while the relatively short follow-up period and missing data from patients treated at other institutions limit the scope of our long-term findings.

We would recommend mechanisms be put in place for this information to be made available for the purpose of quality assurance. The gap between improved local control and survival rates is consistent across multiple randomized studies of preoperative therapy in rectal cancer [5-8]. To improve cancer survival rates, more effective systemic approaches are necessary. Ongoing research into intensive chemotherapy regimens and biologic agents may enhance results in patients with metastatic disease [9].

More recent trials have investigated chemotherapy earlier in the treatment course. The PRODIGE 23 randomized trial employed three months of neoadjuvant chemotherapy (FOLFIRINOX) before CRT and surgery, which significantly improved disease-free survival (76 vs 69%) when compared to preoperative CRT [10].

Total neoadjuvant therapy (TNT) delivers all planned preoperative radiotherapy and chemotherapy prior to surgery. The RAPIDO randomized trial used short-course radiotherapy (SCRT) with 5 Gy x 5 fractions (with an optional boost), followed by neoadjuvant chemotherapy before surgery. This resulted in a reduction in disease-related treatment failure and distant metastases. Unfortunately, this was associated with an increased risk of LRR (12% vs 8%) after five years [11]. The OPRA trial investigated a selective "watch-and-wait" strategy for patients who achieved a clinical full response following CRT and TNT. Organ preservation is achievable in half of the patients with rectal cancer treated with TNT, without an apparent detriment in survival. Three-year TME-free survival was improved when CRT was used prior to neoadjuvant chemotherapy (53 vs 41%) [12].

## Conclusions

Our retrospective study at the GRRCC confirms the efficacy of preoperative CRT in a contemporary cohort. These data may serve as a comparison group as we increasingly employ a TNT approach. In an era of decentralized cancer treatment, where chemotherapy is increasingly being delivered at satellite clinics, accessible information systems should be established to facilitate quality assurance and research.
